# Comparison of the Effectiveness of the Ultrasonic Method and Cone-Beam Computed Tomography Combined with Intraoral Scanning and Prosthetic-Driven Implant Planning Method in Determining the Gingival Phenotype in the Healthy Periodontium

**DOI:** 10.3390/ijerph191912276

**Published:** 2022-09-27

**Authors:** Magdalena Bednarz-Tumidajewicz, Aneta Furtak, Aneta Zakrzewska, Małgorzata Rąpała, Karolina Gerreth, Tomasz Gedrange, Wojciech Bednarz

**Affiliations:** 1 Department of Periodontology, Specialist Outpatient Medical Clinic MEDIDENT in Gorlice, 38-300 Gorlice, Poland; 2 Department of Periodontology, Medical University in Wroclaw, 50-041 Wroclaw, Poland; 3 Department of Pediatric Surgery, Marciniak Hospital, 50-041 Wroclaw, Poland; 4 Department of Risk Group Dentistry, Chair of Pediatric Dentistry, Poznan University of Medical Sciences, 60-812 Poznan, Poland; 5 Department of Orthodontics, Carl Gustav Carus Campus, Technische Universität Dresden, D-01309 Dresden, Germany; 6 Department of Dental Surgery, Medical University in Wroclaw, Krakowska 26 Str., 50-425 Wroclaw, Poland

**Keywords:** ultrasound, cone-beam computed tomography, digital scanning, phenotype, gingival thickness, dental implants, gingival recession, peri-implantitis, periodontitis

## Abstract

The aim of this study was to compare the effectiveness of two diagnostic methods: ultrasonic gingival thickness measurement (UGTM) and cone-beam computed tomography, intraoral scanning by computer-aided design technology with prosthetic-driven implant planning software (CBCT/CAD/PDIP) in determining the gingival phenotype (GP). Thirty periodontally healthy patients were examined. The ultrasonic device Pirop G^®^ with a frequency of 20 MHz and CBCT/CAD/PDIP were used to measure gingival thickness at upper canines and incisors in three points localized midbuccally, namely free gingival thickness (FGT), supracrestal (SGT) and crestal (CGT). Probing depth (PD), clinical attachment level (CAL) and width of keratinized tissue (WKT) were measured using periodontal probe. Intra-examiner and inter-examiner agreement and agreement between methods were evaluated using Bland-Altman analyses. Comparing both methods in the determination of SGT (bias = 0.17 mm, SD = 0.25 mm, *p* < 0.000) and CGT (bias = −0.45 mm, SD = 0.32 mm, *p* < 0.000) 95.0% and 95.6% agreement were found, respectively, and in the FGT range only 93.3% (bias = −0.45 mm, SD = 0.32 mm, *p* < 0.000). The presence of positive correlations between WKT and SGT was shown. A positive correlation between SGT and WKT confirms the purpose of measuring these parameters for the evaluation of the GP. Both the ultrasonic method and cone-beam computed tomography combined with intraoral scanning and prosthetic-driven implant planning method were useful in determining gingival phenotype, however, the ultrasonic method was more accurate for measuring GT.

## 1. Introduction

**Figure 1:**
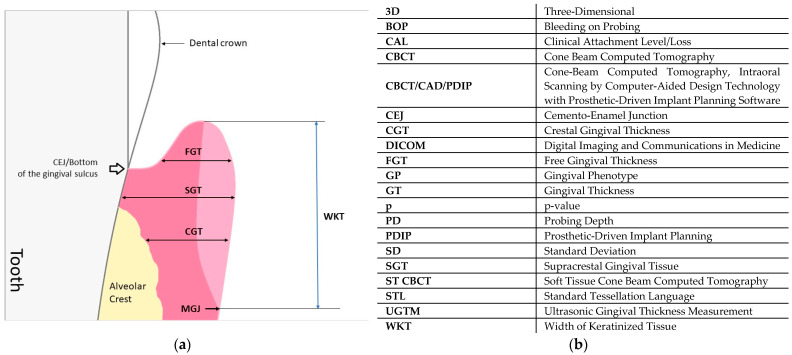
(**a**) Scheme of the periodontal structures and (**b**) abbreviations used in the manuscript.

Gingival phenotype (GP) along with the bone morphotype of the alveolar process determines the type of periodontal phenotype [1]. The gingival phenotype is defined for each dento-gingival unit as a three-dimension volume of the gingiva. The clinical parameters describing GP are gingival thickness and width of keratinized tissue [2] (Figure 1a,b). Clinical evaluation of the gingival phenotype plays an increasingly important role in diagnostics, dental treatment planning, and prognosis for its results [3]. Determination of the gingival phenotype for individual dento-gingival units, instead of the general evaluation of the entire mucogingival complex of the patient, is required before numerous dental procedures [4]. The type of gingival phenotype has a significant impact on the course of wound healing after surgical and regenerative treatment [5]. Proper determination of the gingival phenotype allows for the creation of an optimal treatment plan and may protect against complications in orthodontic, periodontal, prosthetic, and dental implant treatment [6,7,8,9,10,11,12,13]. In the diagnosis of the gingival phenotype, methods based on the shape of the crowns of upper incisors, and based on the transparency of the free gingiva of the upper incisors, the dimension of gingival papillae, and the width of the keratinized tissue are used [14,15].

Gingival transparency methods define the phenotype for the examined dentogingival unit. They are non-invasive, however, rely only on the thickness and density of free gingiva. Therefore, the biometric methods measuring the gingival thickness and the width of the keratinized tissue (WKT) seem to be burdened with a lower risk of measurement error. The measurement of WKT is commonly performed using a periodontal probe calibrated up to 1 mm, while gingival thickness measurement is carried out with the use of invasive, minimally invasive, or non-invasive methods [16,17,18,19,20,21,22,23]. An invasive examination is performed under local anesthesia by puncturing the soft tissues (bone sounding) with a periodontal probe, injection needle, or endodontic instrument [16,17,24]. Radiological examination (parallel profile radiographs, CBCT—cone beam computed tomography) is atraumatic method but associated with receiving a dose of ionizing radiation, whereas optical coherence tomography and ultrasound examination are non-invasive [18,21,22,25,26]. Retraction of lip and tongue from the alveolar processes and teeth during scanning of the patient, which is used in the Soft Tissue Cone Beam Computed Tomography (ST CBCT) technique, allows for very detailed imaging of periodontal soft tissues [12,27,28].

In modern CBCT devices, the risk of radiation is much more reduced [29], therefore, they are widely used in dental diagnostics [22,28,30,31,32]. Moreover, 3D intraoral scanners are also utilized for imaging oral tissues, including teeth and the surface of the mucosa [22,33,34,35,36]. The scans of the gingiva and the mobile mucous membrane, conducted before the augmentation procedures, were superimposed on post-procedure scans, and the existing differences were compared, namely the increase in the volume of soft tissue.

Currently, the obtained images of the surfaces of the teeth, gingiva, and mucosa are saved as STL files. They could be imported into the software, together with the image of tissue received in the CBCT examination, and used in Prosthetic-Driven Implant Planning (PDIP) [22,36,37]. Three-dimensional visualization of oral tissues, based on combining the images of CBCT examination with the results of intraoral scanning, allows for more accurate and precise measurements of gingival thickness as well as the thickness of the labial alveolar bone plate to categorize periodontal phenotype.

The use of an ultrasound device in the measurement of the thickness of periodontal soft tissues and determining the gingival phenotype is a non-invasive method. It effectively eliminates the patient’s discomfort and limitations that arise when using invasive methods which are related to the need for anesthesia use, and causing a temporary increase in the volume of the examined soft tissues [19,38]. However, it requires the experience and calibration of the examiner, especially in terms of achieving optimum probing force. But it is considered an effective and repeatable method [21,39]. The studies confirm the repeatability and efficacy of other measurement methods of gingival tissue thickness [24,35].

Kloukos et al. [24,40] and Gürlek et al. [41] emphasized, that it is impossible to find a gold standard in measuring the gingival thickness, as each method is affected by errors, even the direct measurement method during a surgical procedure using an orthodontic caliper or an Iwanson device. Therefore, assessment of the reliability of the measurement method on the basis of repeatability and reproducibility is crucial.

The study aims to compare the effectiveness of the computed tomography and intraoral 3D camera scanning method (CBCT/CAD) using PDIP^®^ (Prosthetic-driven Implant Planning) software with the ultrasound method (UGTM) in determining the gingival phenotype in the healthy periodontium.

## 2. Materials and Methods

The study was conducted in accordance with the Declaration of Helsinki of 1975 as revised in 2000. The study was approved by the Ethical Committee of the Wroclaw Medical University (resolution no. KB-245/2018). All individuals who participated in the research were informed about the purpose and methods before the study and signed informed consent forms.

The study included 30 volunteer patients, 16 males, and 14 females, aged from 24 to 54 years (mean age amounted to 34 years). The study group was also assessed in the previous research by Bednarz-Tumidajewicz et al. 2020 [22]. The current study is a continuation of the aforementioned research carried out by the same research team. However, in the cited work, clinical and radiological parameters were measured once and it was conducted only by one examiner. The study protocol and division into gingival phenotypes according to the mean values of FGT and SGT by the CBCT/CAD with the PDIP method were also similar. All individuals required diagnostics with the use of CBCT before orthodontic, surgical, and implantoprosthetic treatment. Six dentogingival units in the anterior segment of the maxilla, i.e., canines and incisors were evaluated in each patient (in total 180 dentogingival units were assessed in all individuals). Clinical and radiological examinations were performed in the Specialist Outpatient Medical Clinic MEDIDENT in Gorlice (Poland).

The inclusion criteria for the study were as follows:

(a) no general diseases,
(b) good oral hygiene (Approximal Plaque Index < 15%),
(c) BOP (Bleeding on probing) for the entire oral cavity <10%,
(d) no loss of clinical attachment in the examined areas (Clinical Attachment Level/Loss, CAL = 0),
(e) not using drugs that may affect the structure of periodontal tissues,
(f) no addictions, especially nicotine use/mainly cigarette smoking,
(g) not using removable prosthetic restorations and orthodontic appliances,
(h) no contraindications for radiological examination.

Before the clinical examination, the examiners were trained and calibrated in terms of the appropriate probing force on the soft tissues of the periodontium, in both methods, i.e., measurements performed with the use of a periodontal probe and the Pirop G^®^ ultrasound device.

During the procedure, two researchers (MB-T and WB) carried out, in vitro, measurements of the gingiva thickness of the pig’s jaw placed on an electronic scale, with an accuracy of 0.01 g with a pressure force not exceeding 25 g. After obtaining acceptable accuracy and repeatability of measurements of each examiner, calibration was performed between both examiners. Similarly, each examiner was calibrated for linear measurements of CBCT/CAD/PDIP on the computer monitor. The calibration was considered acceptable when the percentage of agreement according to Bland-Altman analysis was above 90%.

### 2.1. Clinical and Radiological Assessment

Each patient underwent periodontal examination. The probing depth (PD), the Clinical Attachment Level (CAL), and the width of keratinized tissue (WKT) were measured using a periodontal probe (PCP-UNC 15^®^, Hu-Friedy, Chicago, IL, USA) calibrated to 1 mm. Gingival thickness (GT) was measured by two methods: ultrasound (GT_u_) and using CBCT/CAD with PDIP technology (GT_t_). Three points localized midbuccally were designated. Free Gingival Thickness (FGT) was measured at the point exactly in the middle of the distance between the gingival margin and the bottom of the gingival groove. Supracrestal Gingival Thickness (SGT)—in the point located 1 mm apically from the cemento-enamel junction (CEJ). Crestal Gingival Thickness (CGT)—at a distance of 1 mm from the edge of the alveolar bone. The gingival phenotypes were determined by measuring the gingival thickness (GT) using both methods according to the following criteria:

GT ≤ 0.7 mm—thin gingival phenotype,
GT > 0.7 mm ≤ 1.0 mm—medium gingival phenotype,
GT > 1.0 mm—thick gingival phenotype.

To establish repeatability and reproducibility of measurement, two measurements of the tested variables were performed by each examiner (M.B-T, W.B.), with two days intervals between the sessions, and under the same conditions. Clinical measurements were performed at 2.5 times optical magnification.

### 2.2. Ultrasound Examination

**Figure 2:**
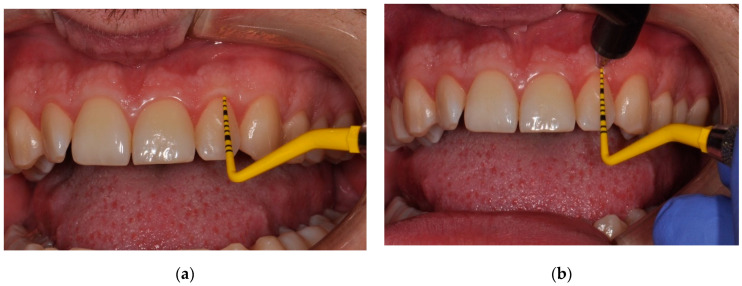
Ultrasonic gingival thickness measurement with the Pirop G^®^ device: (**a**) probing depth measurement, (**b**) supracrestal gingival tissue measurement—the center of the probe head is situated 1 mm apically from CAL.

An ultrasound examination was performed on each patient qualified for the procedure, using a Pirop G^®^ ultrasound device (Echo-Son SA, Pulawy, Poland), with a 20 MHz frequency, and with a probe forehead diameter of 1.7 mm. All parameters were adapted to measure the thickness of the gingiva, palatal mucosa and the mobile mucosa covering the jawbones. The device in the A-scan mode enabled the measurement the tissue thickness at the examined point (Figure 2a,b). The measuring range was 0.2–6 mm. The ultrasonic impulse sent from the transducer at a speed of 1540 m/s, after obtaining perpendicularity in relation to the bone or tooth, returned to the device in time allowing for automatic calculation of the traveled distance that finally resulted in the measurement of the thickness of the soft tissue.

### 2.3. Cone-Beam Computed Tomography/Computer-Aided Design and Prosthetic-Driven Implant Planning

**Figure 3:**
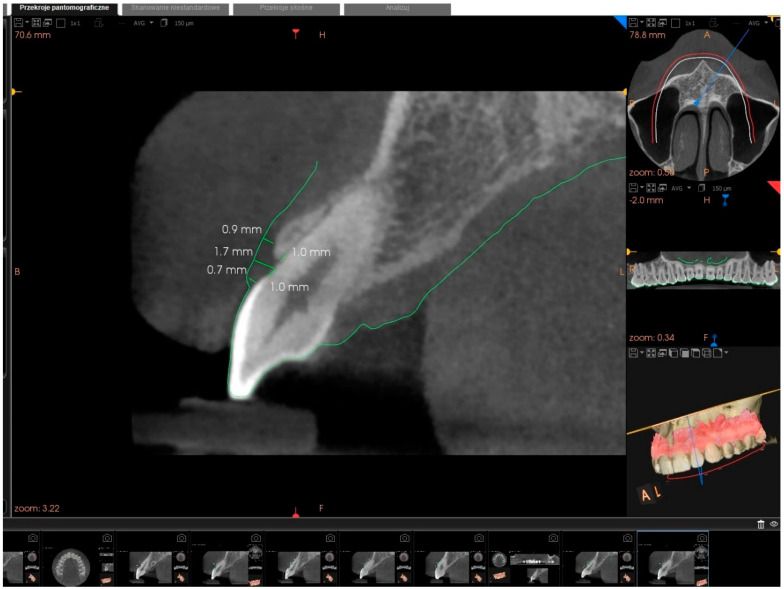
Cone-beam computed tomography, intraoral scanning by computer-aided design technology along with prosthetic-driven implant planning software for FGT, SGT, and CGT measurement.

All the CBCT examinations were performed with the computed tomography device (CS 8100 3D Access, Carestream Dental, Toulouse, France) with the following settings: 90 kV and 3.20 mA for 15 s (voxel size: 150 µm, greyscale: 15 bit, focal spot: 0.6 mm, field of view: 80 × 90 mm). For intraoral scanning 3D CS 3600 intraoral scanner (Carestream Dental, Toulouse, France) was used. The GT measurements were performed with the use of the tomography device and the PDIP software (Carestream Dental, Toulouse, France). The CBCT scans were worked out and developed using the OnDemand 3D software package and saved in DICOM format (digital imaging and communications in medicine). Intraoral optical scan files with images were saved in STL (standard tessellation language). Both files were exported into prosthetic-driven implant planning software for the mentioned measurements and visual analysis. PDIP software allows for automatic integration of the intraoral scan image with the CBCT image. As a result, the surface of soft tissues and teeth is marked with a green line. By setting the gained image in the sagittal section in the midbuccal position, it is possible to measure the linear segments between the points on the surface of the gums (green line) and the alveolar ridge or teeth. The use of lip retractors during CBCT is not necessary using the software (Figure 3).

### 2.4. Statistical Analysis

The results of the study were subjected to statistical analysis. Mean values (x), medians (M), range (min-max), upper and lower quartile (25–75Q) and standard deviations (SD) of the studied continuous parameters were calculated for all groups. Intra-examiner and inter-examiner agreement and agreement between measurement methods were evaluated using Bland-Altman analyses. To assess the accuracy of the measurement, the following were calculated: 95% confidence interval, 95% limits of agreement, percentage of agreement, standard deviation, and statistical significance between measurement methods (Wilcoxon matched-pairs signed rank test). Verification of the hypothesis about the equality of the mean parameters in independent groups was conducted using the ANOVA variance analysis method, or for groups without heterogeneous variance, using the non-parametric U Mann–Whitney’s test, whereas homogeneity of variance was verified using Barlett’s test.

For selected pairs of parameters, a correlation analysis was carried out to calculate the Spearman correlation coefficient. *p* ≤ 0.05 was considered statistically significant. Statistical analysis was performed with the use of computer packages of statistical programs STATISTICA VER. 13 I EPIINFO Ver. 7.2.4.

## 3. Results

Descriptive statistics of clinical and radiological measurements were demonstrated in Table 1. Table 2 and Table 3 show the intra-examiner agreement of measurement performed by first (M.B-T.) and second examiner (W.B.). Table 4 presents the inter-examiner agreement of measurement, and Table 5 shows the comparison between methods in bias. Table 6 shows the mean values of the variables in individual gingival phenotypes according to the division criteria in accordance to the values of FGT_u_, FGT_t_ and SGT_u_.

The first examiner achieved agreement equal to or greater than 95% in the repeatability of FGT, SGT, and CGT measurements by ultrasonic method and only SGT measurement using the CBCT/CAD with PDIP technology method. The results of measurement performed using periodontal probe, excluding CAL, were lower than 95% (PD agreement = 92.2%, WKT agreement = 85%) (Table 2).

The second researcher obtained a similar agreement between first and second measurements in the GT range. On the other hand, for the PD value, the agreement was 97.2% and the WKT value was 93.3% (Table 3).

There was no more than 95% agreement between the values of the parameters assessed by both investigators in FGT (92.8%) using the CBCT/CAD/PDIP technology and the WKT method (92.2%) using the periodontal probe (Table 4).

The mean PD value was 1.23 ± 0.38 mm, and the mean value of KT amounted to 5.37± 1.28 mm (Table 1). The mean values of gingival thickness measured with the use of ultrasound method were: FGT_u_ = 0.98 ± 0.23 mm, SGT_u_ = 1.1 ± 0.25 mm, CGT_u_ = 0.69 ± 0.16 mm, and in CBCT/CAD/PDIP were: FGT_t_ = 0.81 ± 0.18 mm, SGT_t_ = 1.57 ± 0.31 mm, CGT_t_ = 0.88± 0.24 mm. The differences between the mean values of FGT, SGT, and CGT assessed with both methods were statistically significant (*p* < 0.0000). The presence of a weak positive correlation was found between the mean value of WKT and SGT_u_ (r = 0.29, *p* < 0.000), WKT and CGT_u_ (r = 0.25, *p* < 0.000) in UGTM method as well as WKT and SGT_t_ (r = 0.35, *p* < 0.000), WKT and FGT_t_ (r = 0.35, *p* < 0.000). However, no correlation was found between WKT and FGT_u_, and CGT_t_.

Due to the low degree of intra-examiner agreement in both investigators and the high inter-examiner reproducibility of CGT_t_, this parameter was not used to determine the gingival phenotype (Table 2, Table 3 and Table 4). The division into three gingival phenotypes was made according to the criteria of the mean value of SGT_u_, however, no such division was made according to the SGT_t_ value criteria, because of the fact that the division was as follows: thin GP (SGT_t_ ≤ 0.7 mm)—1 dentogingival unit, medium GP (SGT_t_ > 0.7 mm ≤ 1.0 mm)—6 dentogingival units, thick GP (SGT_t_ > 0.7 mm)—173 dentogingival units. In order to compare the determination of gingival phenotypes with both methods, the division criteria was based on the values of FGT_u_ and FGT_t_, despite the lack of > 95% intra-examiner repeatability and inter-examiner reproducibility in the FGT_t_ range.

Comparing both methods in the determination of SGT (bias = 0.17 mm, SD = 0.25 mm, 95% CI = 0.13 mm; 0.21 mm, 95% LOA = −0.33 mm; 0.66 mm, *p* < 0.000) and crestal GT (bias = −0.45 mm, SD = 0.32 mm, 95% CI = −0.50 mm; −0.41 mm, 95% LOA = −1.08 mm; 0.17 mm, *p* < 0.000), 95.0% and 95.6% agreement was found, respectively, and in the FGT range only 93.3% (bias = −0.45 mm, SD = 0.32 mm, 95% CI = −0.50 mm; −0.41 mm, 95% LOA = −1.08 mm; 0.17 mm, *p* < 0.000) (Table 5).

Using the UGTM method in the examined 180 dento-gingival units in 30 patients, based on the FGT_u_ values, 21 sites with a thin phenotype (11.67%) were found, 85 sites with a medium phenotype (47.22%), and 74 sites with the thick phenotype (41.11%). The mutual comparison between the three gingival phenotypes (thin-thick, thin-medium, medium-thick) showed no statistically significant differences in the mean values of WKT. Additionally, there were no differences in mean CGT_u_ values between the medium and thick phenotype (Table 6).

Using the CBCT/CAD with PDIP method, on the basis of the mean FGT_t_ value as the division criteria, all 180 dento-gingival units were divided into 65 sites (36.11%) with a thin phenotype, 101 sites (56.11%) with medium phenotype, 14 sites (7.78%) with a thick gingival phenotype. The comparison between three gingival phenotypes showed statistically significant differences in all examined variables (Table 6).

The division into gingival phenotypes based on the mean SGT_u_ value was as follows: eight sites (4.45%) with thin GP, 58 sites (32.22%) with medium GP, and 114 sites (63.33%) with thick GP. When comparing CGT_u_ and WKT, and describing the thin and medium phenotype, no statistically significant differences were found. Similarly, no statistically significant differences in mean WKT values were noted between the thin and thick phenotype, while the other assessed variables differed significantly. There was a statistically significant difference between the medium and thick phenotype in the mean values of WKT, SGT_u,_ and CGT_u_ (Table 6).

## 4. Discussion

In recent years, biometric methods have been used to determine the gingival phenotype of individual dento-gingival units in order to include the results in dental treatment planning, mainly in orthodontics and implantology. The width of the keratinized tissue, which is the first component of the gingival phenotype, is standardized and based on measurement using the periodontal probe. Biometry of another component, i.e., gingival thickness, was for a long period of time based on invasive bone sounding and considered the gold standard [17,19,42]. However, many authors questioned its accuracy, showing errors at the level of measuring and reading the measurement value [19]. The ultrasound method was also questioned since the researchers emphasized its high inaccuracy and lack of measurement repeatability [43]. But at that time, the appropriate equipment was not available. Therefore, mainly ophthalmic devices were utilized but they were equipped with large probes. Thus, the study site was often random despite its determination. However, the precise determination of the measurement site concerned the invasive method. Some authors have measured GT midbuccally in the middle of keratinized tissue [44], and additionally in the second point on the level of 2 mm apically from the mucogingival junction [17,19]. The GT measurements were also determined at three points, namely 1 mm apically from the gingival margin, coronally to the mucogingival line, and in the midpoint between them [45]. Other researchers studied GT at one point, i.e., 1.5 mm from the gingival margin [42,46,47], 2 mm from the gingival margin [48], or at a point located 3 mm from the gingival margin [49]. However, subsequent studies revealed that the accurate determination of the GT measurement site is also possible in the UGTM method and radiological methods. Stein et al. (2012) [50] using the parallel profile radiograph method studied the thickness of the free gingiva in two points: marginal (G1) and closer to the clinical attachment (G2), supracrestal gingiva attached in three points: at the CEJ level (G3), immediately supracrestally (G4) and between them (G5), and crestal gingiva in a point located directly at the bone margin (G6). Furtak et al. [21] using the UGTM method and Bednarz-Tumidajewicz et al. [22] using CBCT/CAD with PDIP technology measured gingival thickness in 3 points: FGT, SGT, and CGT, similar to this study. The current ultrasound devices are adapted to the examination of gingival thickness and provide high measurement accuracy [11,21,39,43]. According to the literature data the ultrasound devices used in the field of dentistry are in A-mode [24,51,52,53,54], B-mode [37,55], and high frequencies B-mode—40 MHz [56] and 70 MHz in B-mode and Doppler mode acquisitions [57]. B-mode devices are rarely used in dental practice. Research by Izzetti et al. [57] showed that it is even possible to measure gingiva epithelial thickness. B-mode devices are expensive, need extensive experience in examining and interpreting the obtained images from the examiner, what is more, require a lot of chair time and at least 2 weeks of calibration [37,56]. Devices operating in A-mode, where the measurement is performed automatically, seem to be a better solution [13,21,22,54]. The fact that the examination is well tolerated by patients, including children, is also important [54]. The condition for obtaining high repeatability and measurement accuracy, in addition to the precise determination of the measurement site, is the experience of the examiner based on knowledge of the craniofacial anatomy, the mechanism of operation of the ultrasound biometer, and the tactile measurement methodology that requires preclinical calibration [21,39]. Similar conclusions, regarding the effectiveness and repeatability of linear measurements in CBCT imaging and intraoral scanning, were formulated in other studies [35,58,59]. The authors emphasized the importance of resolution, voxel size, field of view size, the use of CAD software that is compatible with an intraoral scanner, and the examiner’s experience in the accuracy of linear measurements [35,60,61,62]. In our study, two investigators received positive results during the calibration process before the beginning of the clinical examinations. The obtained high intra-examiner and inter-examiner agreement concerning gingival thickness measurements using the UGTM method and SGT using CBCT/CAD with the PDIP method should be considered optimal.

The low intra-examiner agreement between the two investigators was observed in the CGT range. It indicated low repeatability of measurements of gingival thickness at this point using CBCT, intraoral scanning method, and PDIP with a high inter-examiner agreement. The FGT_t_ demonstrated better agreement of measurement, but still below 95%. However, due to the high SGT_t_ values and the inability to divide into phenotypic groups according to the criteria assumed in the study, the division into phenotypes was carried out according to the average FGT_t_ values.

A low level of intra-examiner agreement for each investigator and 92.2% of agreement between examiners was revealed in the mean value of WKT. A slightly better agreement, however, also insufficient, was demonstrated in terms of the mean value of PD. It is most likely related to the unavoidable rounding of results to 1 mm, which is the smallest range of the periodontal probe measuring scale. In the case of healthy periodontium of the incisors and canines in the maxilla, 1 mm is the most commonly tested value of PD. On the other hand, in the case of WKT, with different values (in our study: minimum—2 mm, maximum—8 mm), the possibility of making inaccuracies related to rounding the read value was greater. Notwithstanding, despite such a degree of non-compliance, and due to the fact that the WKT value is a component determining the gingival phenotype, these data were used for statistical analysis. Lee et al. 2020 [63] showed the use of intraoral scanning for WKT measurements, which in comparison with using a periodontal probe is more accurate and reliable.

It seems that the demonstrated presence of a positive correlation between the values of SGT measured by the two methods and WKT indicates the accuracy of using these parameters to determine the gingival phenotype for a single dento-gingival unit. The thin gingival phenotype determined by the ultrasound method was characterized by the following values: SGT_u_ = 0.65 mm, WKT = 4.50 mm, whereas medium and thick gingival phenotype with 0.87 mm, 4.92 mm and 1.26 mm, 5.62 mm, respectively. On the other hand, no significant differences in the WKT values between the thin and medium phenotype and the thin and thick phenotype revealed the greater importance of the thickness of the supracrestal gingival tissue in determining the gingival phenotypes, which is consistent with the observations of other authors [15,64].

In our study, despite significant differences in gingival thickness values, there was at least 95% agreement between the assessed methods in the scope of SGT and CGT. When assessing the bias as an indicator of measurement accuracy, the ultrasound method should be considered better than the CBCT/CAD with the PDIP method in determining gingival thickness, as shown in Table 5. Mean SGT_u_ and CGT_u_ values were significantly lower than the corresponding mean SGT_t_ and CGT_t_ values. This is consistent with the observations of Kloukos et al. [40] who assessed the GT of both mandibular central incisors when comparing the ultrasound method and the ST CBCT method in 40 orthodontic patients. The mean value of GT measured 2 mm apically from the gingival margin using the UGTM method amounted to 0.8 mm, and with the use of the CBCT method, it was higher by a minimum of 0.13 mm and a maximum of 0.21 mm. Gkogkos et al. [53] in an ex vivo study performed on 20 porcine cadavers revealed better accuracy in measurement of GT at central mandibular incisors using the Soft Tissue CBCT method than the ultrasonic method. However, the mean GT values measured by the ultrasonic method were not significantly higher than those measured by the ST CBCT method. The comparability of both methods in the measurement of gingival thickness is also confirmed by Sonmnez et al. 2020 [12].

Gürlek et al. [41] in their study compared three methods of GT measurements: transgingival probing using a periodontal probe, CBCT, and UGTM. They showed high compliance between CBCT and transgingival probing. The mean GT values obtained by the ultrasonic device were lower than the other methods, which is consistent with our observations. The mean GT value in 25 patients measured 3 mm apically from gingival margin at upper lateral incisors was 1.59 mm using UGTM, 1.76 mm in transgingival method, and 1.73 mm using CBCT and at canines 1.79 mm, 1.92 mm, 1.90 mm, respectively. In our study SGT point was the most responding point to Gürlek et al. study [37]. The mean SGT values at all examined dentogingival units were 1.11 mm (minimum 0.60, maximum 1.90 mm) using UGTM and 1.57 mm (minimum 0.63 mm, maximum 2.45 mm) using CAD/CBCT and PDIP method.

Couso-Queiruga et al. [35] confirmed the comparability of the measurements of gingival thickness using both methods, i.e., invasive direct measurement and this using digital scanning with images saved in STL and CBCT files in DICOM files. On the other hand, Kloukos et al. [24], during a comparison of four methods of measuring gingival thickness, indicated that the ultrasound method gives higher results than the transgingival probing using the periodontal probe with a silicon stopper, but is similar when using an acupunctural needle. The authors concluded that there was a lack of a gold standard for measuring gingival thickness, but it seems that the ultrasound method may currently be recommended for everyday use in dental practice.

The limitation of this study was the small group of patients and the lack of a gold standard to which the gingival thickness results could be compared. The other limitation was the use of measuring devices where the accuracy was technologically limited to 0.01 mm in UGTM and to 0.1 mm in CBCT/CAD/PDIP, and to 1 mm when using the periodontal probe. An additional limitation was the failure to apply a histochemical test using Lugol’s solution before measuring the width of keratinized tissue.

## 5. Conclusions

Considering the limitations of this study, it should be stated that both the ultrasonic method and cone-beam computed tomography combined with intraoral scanning and prosthetic-driven implant planning method were useful in determining gingival phenotype, however, the ultrasonic method was more accurate for determining gingival thickness (GT). The presence of a positive correlation between the mean values of supracrestal gingival thickness (SGT) and width of keratinized tissue (WKT) confirms the validity of the measurement of these clinical parameters in determining the gingival phenotype. 

## Figures and Tables

**Table 1:** Descriptive statistics of clinical and radiological measurements in millimeters (mm).

Variables	*n*	Mean	Median	Minimum	Maximum	Lower Quartile	Upper Quartile	SD
FGT_u_	180	0.98	0.95	0.43	1.63	0.83	1.10	0.23
SGT_u_	180	1.11	1.11	0.60	1.90	0.92	1.30	0.25
CGT_u_	180	0.69	0.69	0.32	1.36	0.59	0.76	0.16
FGT_t_	180	0.81	0.80	0.50	1.90	0.70	0.90	0.18
SGT_t_	180	1.57	1.56	0.63	2.45	1.38	1.74	0.31
CGT_t_	180	0.88	0.90	0.30	1.70	0.70	1.00	0.24
PD	180	1.23	1.00	1.00	2.00	1.00	1.38	0.38
CAL	180	0.00	0.00	0.00	0.00	0.00	0.00	0.00
WKT	180	5.37	5.25	2.00	8.50	4.00	6.38	1.28

FGT_u_—free gingival thickness—ultrasonic gingival thickness measurement (UGTM), SGT_u_—supracrestal gingival thickness UGTM, CGT_u_—crestal gingival thickness UGTM, FGT_t_—free gingival thickness Cone-Beam Computed Tomography/Computer-Aided Design/Prosthetic-Driven Implant Planning (CBCT/CAD/PDIP), SGT_t_—supracrestal gingival thickness CBCT/CAD/PDIP, CGT_t_—crestal gingival thickness CBCT/CAD/PDIP, PD—probing depth, CAL—clinical attachment level, WKT—width of keratinized tissue, SD—standard deviation.

**Table 2:** Intra-examiner agreement of measurement performed by the first examiner (M.B-T.).

Variables	Bias	−95% CI	95% CI	SD	−95% LOA	95% LOA	% Agreement	*p*
FGT_u_	0.00	0.00	0.01	0.03	−0.05	0.06	95.00	0.032
SGT_u_	0.00	−0.00	0.01	0.03	−0.06	0.06	95.00	0.073
CGT_u_	−0.01	−0.01	−0.00	0.03	−0.06	0.05	98.90	0.036
FGT_t_	0.00	−0.00	0.00	0.03	−0.05	0.05	92.80	0.782
SGT_t_	0.04	0.03	0.05	0.08	−0.12	0.20	100.00	0.000
CGT_t_	−0.00	−0.01	0.00	0.04	−0.08	0.07	85.60	0.240
PD	0.00	−0.04	0.04	0.28	−0.55	0.55	92.20	0.999
CAL	0.00	0.00	0.00	0.00	0.00	0.00	100.00	1.000
WKT	−0.04	−0.09	0.02	0.39	−0.79	0.71	85.00	0.179

FGT_u_—free gingival thickness UGTM, SGT_u_—supracrestal gingival thickness UGTM, CGT_u_—crestal gingival thickness UGTM, FGT_t_—free gingival thickness CBCT/CAD/PDIP, SGT_t_—supracrestal gingival thickness CBCT/CAD/PDIP, CGT_t_—crestal gingival thickness CBCT/CAD/PDIP, PD—probing depth, CAL—clinical attachment level, WKT—width of keratinized tissue, 95% CI—95% confidence interval, SD—standard deviation of Bias, 95% LOA—95% limit of agreement, % agreement—percentage of agreement, *p*—statistical significance Wilcoxon matched-pairs signed rank test.

**Table 3:** Intra-examiner agreement of measurement performed by the second examiner (W.B.).

Variables	Bias	−95% CI	95% CI	SD	−95% LOA	95% LOA	% Agreement	*p*
FGT_u_	0.00	−0.00	0.01	0.03	−0.07	0.07	96.70	0.771
SGT_u_	0.19	0.15	0.25	0.35	−0.50	0.89	95.00	0.000
CGT_u_	0.00	−0.00	0.01	0.04	−0.07	0.07	97.80	0.938
FGT_t_	−0.00	−0.03	0.02	0.15	−0.29	0.29	90.60	0.957
SGT_t_	0.01	−0.00	0.01	0.06	−0.12	0.13	100.00	0.192
CGT_t_	−0.00	−0.01	0.00	0.04	−0.09	0.08	80.60	0.128
PD	0.00	−0.02	0.03	0.17	−0.32	0.33	97.20	0.656
CAL	0.00	0.00	0.00	0.00	0.00	0.00	100.00	1.000
WKT	0.01	−0.03	0.05	0.26	−0.49	0.52	93.30	0.565

FGT_u_—free gingival thickness UGTM, SGT_u_—supracrestal gingival thickness UGTM, CGT_u_—crestal gingival thickness UGTM, FGT_t_—free gingival thickness CBCT/CAD/PDIP, SGT_t_—supracrestal gingival thickness CBCT/CAD/PDIP, CGT_t_—crestal gingival thickness CBCT/CAD/PDIP, PD—probing depth, CAL—clinical attachment level, WKT—width of keratinized tissue, 95% CI—95% confidence interval, SD—standard deviation of Bias, 95% LOA—95% limit of agreement, % agreement—percentage of agreement, *p*—statistical significance Wilcoxon matched-pairs signed rank test.

**Table 4:** Inter-examiner agreement of measurement between two examiners.

Variables	Bias	−95% CI	95% CI	SD	−95% LOA	95% LOA	% of Agreement	*p*
FGT_u_	−0.01	−0.01	−0.00	0.04	−0.08	0.07	98.30	0.007
SGT_u_	−0.08	−0.10	−0.05	0.19	−0.44	0.29	95.00	0.000
CGT_u_	0.02	0.01	0.02	0.03	−0.04	0.08	96.10	0.000
FGT_t_	0.01	−0.00	0.02	0.07	−0.14	0.15	92.80	0.201
SGT_t_	0.01	0.00	0.02	0.06	−0.11	0.13	95.60	0.006
CGT_t_	0.00	−0.00	0.01	0.03	−0.06	0.06	96.10	0.537
PD	−0.01	−0.05	0.03	0.26	−0.52	0.50	95.60	0.669
CAL	0.00	0.00	0.00	0.00	0.00	0.00	100.00	1.000
WKT	0.18	0.10	0.26	0.54	−0.88	1.24	92.20	0.000

FGT_u_—free gingival thickness UGTM, SGT_u_—supracrestal gingival thickness UGTM, CGT_u_—crestal gingival thickness UGTM, FGT_t_—free gingival thickness CBCT/CAD/PDIP, SGT_t_—supracrestal gingival thickness CBCT/CAD/PDIP, CGT_t_—crestal gingival thickness CBCT/CAD/PDIP, PD—probing depth, CAL—clinical attachment level, WKT—width of keratinized tissue, 95% CI—95% confidence interval, SD—standard deviation of Bias, 95% LOA—95% limit of agreement, % agreement—percentage of agreement, *p*—statistical significance Wilcoxon matched-pairs signed rank test.

**Table 5:** Agreement between methods in the measurement of gingival thickness.

Ultrasound Method	CBCT/CAD/PDIP	Bias	−95% CI	95% CI	SD	−95% LOA	95% LOA	% Agreement	*p*
FGT_u_	FGT_t_	0.17	0.13	0.21	0.25	−0.33	0.66	93.30	0.000
SGT_u_	SGT_t_	−0.45	−0.50	−0.41	0.32	−1.08	0.17	95.00	0.000
CGT_u_	CGT_t_	−0.19	−0.22	−0.15	0.25	−0.68	0.30	95.60	0.000

FGT_u_—free gingival thickness UGTM, SGT_u_—supracrestal gingival thickness UGTM, CGT_u_—crestal gingival thickness UGTM, FGT_t_—free gingival thickness CBCT/CAD/PDIP, SGT_t_—supracrestal gingival thickness CBCT/CAD/PDIP, CGT_t_—crestal gingival thickness CBCT/CAD/PDIP, 95% CI—95% confidence interval, SD—standard deviation of Bias, 95% LOA—95% limit of agreement, % agreement—percentage of agreement, *p*—statistical significance Wilcoxon matched-pairs signed rank test.

**Table 6:** Variables assessed in individual gingival phenotypes according to the criteria of dividing the mean values of FGT_u_, FGT_t_ and SGT_u_ in mm.

Gingival Phenotype	Variables	*N*	Thin GP	Thin GP/Medium GP *p*	*N*	Medium GP	Medium GP/Thick GP *p*	*N*	Thick GP	Thin GP/Thick GP *p*
Acc to FGT_u_	FGT_u_	21	0.67 (0.60 ÷ 0.69)		84	0.88 ± 0.07 0.89 (0.82 ÷ 0.94)		75	1.19 ± 0.17 1.13 (1.06 ÷ 1.35)	
	SGT_u_	21	0.85 (0.72 ÷ 1.00)	0.000 *	84	1.10 ± 0.23 1.12 (0.93 ÷ 1.27)	0.000 *	75	1.20 ± 0.25 1.23 (0.98 ÷ 1.37)	0.009 *
	CGT_u_	21	0.59 (0.47 ÷ 0.72)	0.013 *	84	0.70 ± 0.16 0.69 (0.61 ÷ 0.76)	0.009 *	75	0.70 ± 0.15 0.71 (0.62 ÷ 0.77)	0.823
	WKT	21	5.00 (4.00 ÷ 6.25)	0.759	84	5.36 ± 1.26 5.25 (4.00 ÷ 6.25)	0.712	75	5.40 ± 1.31 5.25 (4.00 ÷ 6.50)	0.858
Acc to SGT_u_	FGT_u_	8	0.69 (0.62 ÷ 0.88)	0.012 *	58	0.96 ± 0.24 0.92 (0.82 ÷ 1.88)	0.145	114	1.01 ± 0.22 0.98 (0.86 ÷ 1.12)	0.001 *
	SGT_u_	8	0.65 (0.64 ÷ 0.66)		58	0.87 ± 0.08 0.88 (0.80 ÷ 0.95)		114	1.26 ± 0.16 1.27 (1.14 ÷ 1.37)	
	CGT_u_	8	0.55 (0.41 ÷ 0.66)	0.125	58	0.62 ± 0.14 0.63 (0.52 ÷ 0.72)	0.000 **	114	0.74 ± 0.15 0.73 (0.64 ÷ 0.81)	0.001 *
	WKT	8	4.50 (3.50 ÷ 6.50)	0.870	58	4.92 ± 1.22 5.00 (4.00 ÷ 5.75)	0.000 **	114	5.62 ± 1.22 6.00 (5.00 ÷ 6.50)	0.245
Acc to FGT_t_	FGT_t_	65	0.65 ± 0.06 0.70 (0.60 ÷ 0.70)		101	0.85 ± 0.08 0.82 (0.80 ÷ 0.90)		14	1.19 (1.10 ÷ 1.23)	
	SGT_t_	65	1.37 ± 0.25 1.38 (1.25 ÷ 1.58)	0.000 **	101	1.63 ± 0.26 1.58 (1.45 ÷ 1.80)	0.000 *	14	1.96 (1.80 ÷ 2.23)	0.000 *
	CGT_t_	65	0.80 ± 0.22 0.80 (0.67 ÷ 0.92)	0.009 **	101	0.90 ± 0.25 0.90 (0.70 ÷ 1.00)	0.004 *	14	1.09 (0.90 ÷ 1.25)	0.000 *
	WKT	65	4.95 ± 1.23 5.00 (4.00 ÷ 6.00)	0.009 **	101	5.48 ± 1.26 5.25 (4.25 ÷ 6.50)	0.005 *	14	6.50 (6.00 ÷ 7.00)	0.000 *

Depending on the distribution the data is presented as mean ± standard deviations or/and as median (lower quartile ÷ upper quartile). Thin GP—GT ≤ 0.7 mm, Medium GP—GT > 0.7 mm ≤ 1.0 mm, Thick GP—GT > 1.0 mm, FGT_u_—free gingival thickness Ultrasonic Gingival Thickness Measurement (UGTM), SGT_u_—supracrestal gingival thickness UGTM, CGT_u_—crestal gingival thickness UGTM, FGT_t_—free gingival thickness Cone-Beam Computed Tomography/Computer Aided Design with Prosthetic-Driven Implant Planning (CAD/CBCT with PDIP), SGT_t_—supracrestal gingival thickness CAD/CBCT with PDIP, CGT_t_—crestal gingival thickness CBCT/CAD with PDIP, WKT—width of keratinized tissue, *p*—statistical significance, *—test U Mann-Whitney, **—test ANOVA, acc—according.

## Data Availability

The data presented in this study are available on request from the main author. The data are not publicly available due to the protection of personal patients data.
